# Macrofilaricidal Activity, Acute and Biochemical Effects of Three Lichen Species Found on Mount Cameroon

**DOI:** 10.1155/2022/1663330

**Published:** 2022-01-07

**Authors:** Thierry Roland Kang, Jerome Nyhalah Dinga, Ayuk Elizabeth Orock, Elvis Monya, Moses Njutain Ngemenya

**Affiliations:** ^1^Department of Biochemistry and Molecular Biology, Faculty of Science, University of Buea, P.O. Box 63 Buea, South West Region, Cameroon; ^2^Department of Environmental Science, Faculty of Science, University of Buea, P.O. Box 63 Buea, South West Region, Cameroon; ^3^Department of Medical Laboratory Sciences, Faculty of Health Sciences, University of Buea, P.O. Box 63 Buea, South West Region, Cameroon

## Abstract

Onchocerciasis is a parasitic infection affecting a relatively small population globally but has very devastating pathological outcomes. Ivermectin and recently moxidectin are the only drugs approved for clinical management of the disease, both of which have several limitations. In particular, they are efficacious against microfilariae (microfilaricidal) with no activity against adult worms (nonmacrofilaricidal). Promising anthelmintic activity has been reported in some lichens. This study investigated three lichens, *Usnea articulata*, *Parmotrema tinctorum*, and *Heterodermia obscurata*, found on Mount Cameroon, for potential macrofilaricidal activity. Organic extracts were screened for anti-Onchocerca activity against *Onchocerca ochengi* isolated from cattle skin using worm motility and MTT formazan assays. Toxicity of highly active extracts was investigated on monkey kidney epithelial (LLCMK2) cells and in BALB/c mice (2000 mg/kg body weight) including effects on liver enzymes. The methanol extract of *P. tinctorum* (Pam_met_) was the most active against adult male worms (IC_50_ = 8.1 *μ*g/mL) with the highest selectivity index (SI = 21.3). *U. articulata* was the most active against the adult female (IC_50_ = 36.3 *μ*g/mL) but had a low SI value (3.4). No mortality and no adverse effects were recorded in the acute toxicity test. These two most active extracts had no significant effect on liver enzymes, alanine aminotransferase, and aspartate (*P* values < 0.05), but a high AST : ALT ratio (2.59) for Pam_met_ indicates likely reversible adverse hepatic toxicity. The high macrofilaricidal activity and selectivity of *P. tinctorum* suggest it is a potential source of new macrofilaricides which should be further investigated to identify its bioactive constituents.

## 1. Introduction

Onchocerciasis (river blindness), caused by *Onchocerca volvulus*, an infectious filarial worm, features among the World Health Organization's neglected tropical diseases [1]. The infection is prevalent in a relatively small geographical area (Tropical Africa 99% and 1% in parts of Middle East, Central and South America) and the population at risk is small (160 million) compared to common infectious diseases. In spite of this, the disease has devastating pathologies which include severe dermatitis, pruritis, and of great medical importance, visual impairment, and subsequent blindness in the absence of early chemotherapeutic treatment [2, 3]. It therefore has a high negative economic impact particularly in riverine populations engaged principally in farming.

Vector control programmes later complemented by community-directed treatment with ivermectin have made significant achievements in reducing the burden and negative impact of the disease, but onchocerciasis is yet to be eliminated [3]. Chemotherapy remains the main tool against the active disease but faces two challenges, namely, lack of suitable anti-Onchocerca drugs and the threat of emerging resistance to ivermectin, which until recently is the only recommended drug for treatment of the disease in the last three decades [4]. Furthermore, ivermectin, though very effective, exerts its effect only against the microfilariae with no killing of the adult worms even after prolonged treatment with repeated doses [5] and produces severe adverse events in areas of *Loa loa* coendemicity. Recently, moxidectin, a chemical analogue of ivermectin, was approved for clinical use against onchocerciasis but is also microfilaricidal and has potential serious toxicity [6]. Hence, the need for new efficacious and safe anti-Onchocerca agents remains high.

Efforts in the discovery of anti-Onchocerca agents especially macrofilaricides have included screening of natural products with promising results [7]. Of interest are lichens which have not been extensively investigated for antihelminthic activity. Prabhu and Sudha [8] reported significant anthelminthic activity of *Heterodermia boryi* macrolichen against Indian earthworm *Pheretima posthuma*. Similar results were reported by Kumar et al. [9], against the same worms. Hence, this study screened crude extracts of three lichens *Usnea articulata* (L.) Hoffm, *Parmotrema tinctorum* (Despr. ex Nyl.) Hale, and *Heterodermia obscurata* (Nyl.) Trevis, Nuovo Giorn, not previously tested for anti-Onchocerca activity, using the *in vitro* model, *Onchocerca ochengi*, a known close relative of *O. volvulus*. The cytotoxicity and acute toxicity of the active lichens were also investigated. In further studies, compounds in the active extracts will be isolated and investigated in view of finding potential new efficacious drugs for treatment of onchocerciasis.

## 2. Materials and Methods

### 2.1. Extract Preparation and Phytochemical Analysis

The three lichens, *Usnea articulata* (L.) Hoffm, *Parmotrema tinctorum* (Despr ex Nyl.) Hale, and *Heterodermia obscurata* (Nyl.) Trevis, Nuovo Giorn, were collected in September 2018, from Mount Cameroon and identified by Dr. Ayuk Elizabeth Orock, a Lichenologist in the Department of Botany, Faculty of Science, University of Buea. The extracts were prepared similarly as described by Bate et al. [10]. Briefly, the lichens were chopped, air-dried under shade for four weeks, and ground into powder. Each powder was then macerated sequentially in hexane followed by methanol and the filtrate concentrated by rotary evaporation (at 68°C and 65°C for hexane and methanol, respectively), dried, weighed, and stored at 4°C in a refrigerator until tested. Phytochemical analysis was performed to determine chemical classes of secondary metabolites present as described [11].

### 2.2. Determination of Anti-Onchocerca Activity

Complete culture medium (CCM) was prepared using RPMI 1640 media containing sodium bicarbonate. It was supplemented with 25 mM HEPES, 0.3 g *γ*-irradiated L-glutamine powder, 5% newborn calf serum (NBS), 200 units/mL penicillin, 200 *μ*g/mL streptomycin, and 0.25 *μ*g/mL amphotericin B; pH 7.4. Incomplete culture medium (ICM) did not contain NBS. All reagents used were from Sigma-Aldrich. All experiments were done twice.

#### 2.2.1. Assays on Adult Worms

This was done as described [12] with some modifications. Briefly, fresh pieces of infected umbilical cattle skin bought from slaughterhouses in Douala and Buea, Cameroon, were washed and sterilized in a sterile lamina flow hood. Nodules were carefully excised, and the recovered worm masses were submerged in 2 mL complete culture medium in 12-well culture plates (NUNC, USA). The plates were incubated overnight at 37°C, 5% CO_2_ in HERACELL-150i incubator (USA), during which time male worms emerged from the masses into the medium and females remained within. Thereafter, the viability of worms and sterility of cultures were evaluated using an inverted microscope (Nikon Eclipse TS100, China) prior to testing and 1 mL of CCM was added in each well containing worm. A primary screen was done to eliminate inactive extracts. Each extract (25 mg/mL stock in dimethyl sulfoxide, DMSO) diluted in CCM to 4x final test concentration was added (1 mL) in triplicate to an adult worm in 3 mL CCM per well giving final concentration of 500 *μ*g/mL. Positive (10 *μ*M auranofin) and negative control (2% DMSO in RPMI) were included. The plate was incubated for 5 days same as above, and male worm viability was assessed using an inverted microscope based on worm motility; inhibition of motility was scored as follows: 100% (no motility), 75% (only head or tail shaking occasionally), 50% (whole worm motile, but sluggish), 25% (only little change in motility), and 0% (no observable change in motility). Activity against female worms was assessed by visual estimation of percentage inhibition of formazan formation following incubation of the worm masses in 500 *μ*L of 0.5 mg/mL MTT (3-(4,5-dimethylthiazol-2-yl)-2,5-diphenyltetrazolium bromide) for 30 min [13]; activity scores ranged from 100% parasite killing (no blue formazan coloration), 90%, 75%, 50%, 25%, to 0% (entire worm appears blue as in negative control). Extract activity based on worm motility or formazan formation was graded as follows: active ≥ 90% inhibition; moderately active 50–89% and inactive < 50% inhibition. A secondary screen was done for extracts with 100% activity in the primary screens by repeating the experiment at serial dilutions of eight concentrations (3.91 to 500 *μ*g/mL), to determine the IC_50_ values [14].

#### 2.2.2. Assays on Microfilariae

These were performed similarly as the assays on adult worms described in detail in previous studies [13, 14] with monkey kidney epithelial (LLCMK2, ATCC, Virginia, USA) cells as a feeder layer. Briefly, the cells were proliferated in CCM until fully confluent, the medium decanted, and cells dislodged with 0.125% trypsin and 0.5 mM EDTA in ICM and then centrifuged twice (560g × 10 minutes). The cell suspension was transferred into 96-well microtitre culture plates (100 *μ*L/well) and incubated as above and grown until fully confluent. Microfilariae (mf) were isolated from cattle skin as described in detail [13, 14], suspended in CCM, and distributed in a 96-well plate (about 15 mfs/100 *μ*L of CCM/well) containing the LLCMK2 cell layer, and their viability and sterility were ascertained for 24 h prior to addition of extracts. Each extract solution was tested in duplicate wells at 500 *μ*g/mL. Positive and negative controls were included followed by incubation as above. Mf viability was scored daily by mean motility as above for 5 days. A secondary screen was done similarly [14] as for the adult worms to determine the IC_50_ value of one extract only.

### 2.3. Cytotoxicity Test

This was also done using monkey kidney epithelial cell [15]. Briefly, the cells were processed and grown to confluence as described above in a 96-well flat-bottom plate in duplicate. The medium was replaced with fresh CCM, and extracts were added at final concentration of 3.9063 to 500 *μ*g/mL. Positive and negative controls were included followed by incubation for 5 days same as above. The medium was discarded, and wells were decolorised by shaking with ICM (IKA Labortechnik KS125 basic shaker) at 600 rpm for 5 minutes twice. MTT (100 *μ*L of 1 mg/mL in ICM) was added, and the plate was incubated as above for 2 hours. Then, the medium was discarded, 100 *μ*L of DMSO was added to dissolve formazan precipitate, and the plate was shaken gently to mix well contents. Well optical densities were read at 595 nm, and percentage inhibition was calculated using the formula:

(1) $$\begin{array}{c} \%\text{Inhibition}=\frac{\text{OD of Negative Control}-\text{OD of Extract}}{\text{OD of negative control }}\times 100. \end{array}$$

### 2.4. Acute Oral Toxicity Test

This was done for the most active extracts on the adult worms, the hexane extract of *U. articulata*, and methanol extract of *P. tinctorum* each in 2% DMSO, as described [15, 16]. Guidelines of the Organization for Economic Cooperation and Development version 423 were followed and ethical approval was obtained from the Institutional Animal Care and Use Committee, University of Buea (UB-IACUC N^O^ 014/2019). Briefly, fifteen BALB/c mice comprising one control and two test groups of five each (9 weeks old, two females and three males per group) from the animal house of the study laboratory were acclimatised for one week. One animal per test group was fasted overnight with water provided and administered 2000 mg/kg body weight of one extract by oral gavage and further observed [15]. With no death or acute adverse toxicity after 24 hours, the other four test animals were fasted and treated the same. All animals were observed for gross changes and signs of adverse toxicity for 14 days. Then, animals were fasted overnight and anaesthetized with ketamine/xylazine (90/10 mg/kg), and blood was collected by retroorbital bleeding. The blood was coagulated for 30 minutes and centrifuged (2200 rpm × 15 minutes), and the activity of liver enzymes, alanine aminotransferase, and aspartate aminotransferase was measured using Biorex Diagnostic Kits (United Kingdom), following the manufacturer's instructions.

### 2.5. Data Analysis

Filaricidal activity data were analyzed using GraphPad Prism 5.0 software (USA) to obtain IC_50_ of active extracts. The logarithm of the extract concentration was plotted against activity, to obtain a nonlinear regression curve-fitting and a variable slope sigmoidal dose-response curve. CC_50_ (concentration at which 50% of cells were dead) was derived from a plot of % inhibition of viability against log extract concentration generated using the same software. Enzyme activities were analyzed using the same software, and an unpaired two-tailed *t*-test was used to check for any significant difference (*P* < 0.05) between the control and test groups.

## 3. Results

### 3.1. Yield and Phytochemical Composition of Extracts

The methanol extract of *P. tinctorum* had the highest yield (12.9%) while the methanol extract of *H. obscurata* had the lowest yield (1.1%), Table 1. Saponins, steroids, flavonoids, and tannins were the common and relatively abundant classes of secondary metabolites in the crude extracts of the lichens. Alkaloids were detected only in hexane extract of *U. articulata* (USa_hex_).

### 3.2. Anti-Onchocerca Activity of Extracts

**Figure 1:**
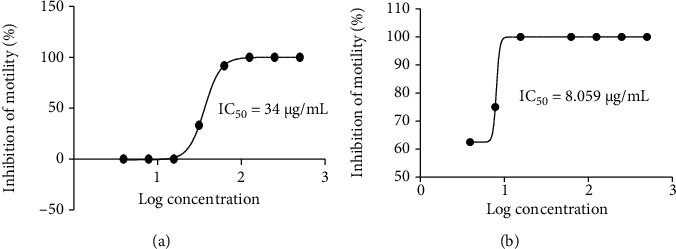
Dose-dependent activity of lichen extracts against *Onchocerca ochengi* adult worms. (a) Hexane extract of *U. articulata* against female worms. (b) Methanol extract of *P. tinctorum* against male worms.

All 6 extracts screened at 500 *μ*g/mL were active against adult male and female *O. ochengi* worms with 100% inhibition in both cases in the primary screen. In the secondary screen, the IC_50_ values ranged from 8.1 to 52.4 *μ*g/mL on adult male worms; the methanol extract of *P. tinctorum* (Pam_met_) was the most active with the IC_50_ value of 8.1 *μ*g/mL. IC_50_s on adult female worms ranged from 36.3 to 115.2 *μ*g/mL; the hexane extract of *U. articulata* (USa_hex_) was the most active with an IC_50_ value of 36.3 *μ*g/mL (Table 2). The extracts showed dose-dependent activity as illustrated for the most active extracts in Figure 1, with an overall higher activity against adult male worms. When the IC_100_ was considered, both extracts of *P. tinctorum* (Pam_hex_ and Pam_met_) were the most active against adult male worms (IC_50_ = 15.6 *μ*g/mL), and both extracts of *U. articulata* (USa_hex_ and USa_met_) were the most active against adult female worms (IC_50_ = 125 *μ*g/mL), showing consistency in activity (Table 2).

On microfilariae, all the 6 extracts screened at 500 *μ*g/mL also showed 100% anti-Onchocerca activity. IC_50_ against microfilariae was determined only for the methanol extract of *Usnea articulata* (USa_met_) giving a value of 101.8 *μ*g/mL.

### 3.3. Toxicity of Extracts

#### 3.3.1. Cytotoxicity

Five extracts had CC_50_ values greater than the cut-off point for lack of cytotoxicity (CC_50_ > 30 *μ*g/mL) [17], and one extract (Het_hex_) had a CC_50_ value lower than 30 *μ*g/mL indicating mild cytotoxicity (Table 2). All extracts showed relatively low selectivity with SI values ranging from 1.08 to 3.89 for both adult male and female worms except the methanol extract of *P. tinctorum* (Pam_met_) which showed a high selectivity index (21.33). Extracts of *H. obscurata* were more toxic to the mammalian kidney cells with SI values < 1. On *O. ochengi* microfilariae, USa_met_ recorded an SI value of 1.25.

#### 3.3.2. Acute Toxicity

The most active extracts, Pam_met_ and USa_hex_, on *O. ochengi* adult male and female worms, respectively, showed no sign of acute toxicity at 2000 mg/kg body weight in Balb/c mice 14 days posttreatment. The animals were sluggish within the first 2 hours postdosing, but no mortality was recorded at day 14. When compared with control mice, intake of food and water were similar. Average weights of mice increased in both control and test groups, but the difference between the groups on day 14 was not statistically significant (*P* = 0.43). No change in physical appearance, behavior, fur, skin, and mucous membranes and physical activity was observed.

#### 3.3.3. Effects of Extracts on Liver Enzymes

**Figure 2:**
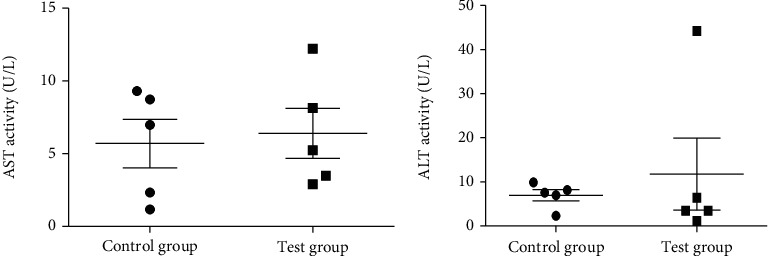
Effect of 2000 mg/kg hexane extract of *Usnea articulata* on mouse liver enzyme activity. AST: aspartate aminotransferase (*P* = 0.77860); ALT: alanine aminotransferase (*P* = 0.5943).

In order to compare the effect on liver enzymes of the hexane extract of *U. articulata* (USa_hex_), which was the most active against female worms, an unpaired two-tailed *t*-test was done for aspartate aminotransferase (AST) at 95% confidence interval (*P* < 0.05) because both control and test groups had the same variances. This was also done for alanine aminotransferase (ALT) with Welch's correction because the variances were not the same. There was no significant difference between the control (2% DMSO) and test (USa_Hex_) groups with *P* values of 0.77860 and 0.5943 for AST and ALT, respectively, as shown in Figure 2. The AST : ALT ratio of mean enzyme activity for the test group was =0.54, indicating no adverse effect in the mouse liver given it is <1.

**Figure 3:**
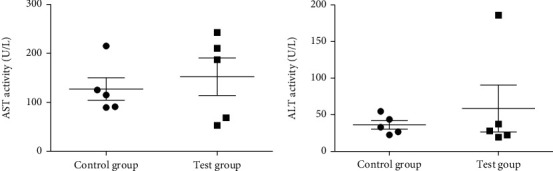
Effect of 2000 mg/kg hexane extract of *Parmotrema tinctorum* on mouse liver enzyme activity. AST: aspartate aminotransferase (*P* = 0.59); ALT: alanine aminotransferase (*P* = 0.52).

Also, no significant difference was found between the control and mice treated with Pam_met_ for body weight (*P* = 0.28), and enzyme activities with *P* values of 0.59 and 0.52 for AST and ALT, respectively, as shown in Figure 3. However, the AST : ALT ratio was 2.59 indicating adverse effect in the liver.

## 4. Discussion

Chemotherapy of onchocerciasis remains a challenge given the very limited number of available filaricides. This study screened extracts of three lichens as a potential source of filaricidal molecules considering their wide-ranging antimicrobial and demonstrated anthelmintic activity [8]. This study recorded high anti-Onchocerca activity for the methanol extract of one lichen, *P. tinctorum*, with high selectivity for the adult male worm. Also, moderate activity was shown by the hexane extract of *U. articulata* against the adult female with a relatively low selectivity (Table 2). This is the first report of the anti-Onchocerca activity of the studied lichens collected from Mount Cameroon.

All the extracts were active against adult worms with IC_50_ values below 100 *μ*g/mL except for one (methanol extract of *H. obscurata*). Overall, the lichen extracts were more active against the adult male worm (lower IC_50_s) than the female. This difference may be due to the smaller size of the male worm which permits greater penetration and bioavailability in the worm tissue of the bioactive secondary metabolites in the extracts, or action via target(s) uniquely present in the male which needs to be further investigated. The promising properties of *P. tinctorum* warrant further investigation. This may yield an efficacious macrofilaricide which upon killing the male worm will prevent fertilization of the female and hence abolish production of microfilariae. The resultant effect will be a marked improvement in patients' welfare since microfilariae are largely responsible for the devastating pathology of onchocerciasis. Partial screening of the extracts on microfilariae also revealed activity against this worm stage indicating potential macro- and microfilaricidal activity in the tested lichens. Further studies will be done to fully assess their microfilaricidal activity. A study of *P. tinctorum* including other Parmotrema species found in Malaysia reported weak antibacterial activity for this lichen, contrary to two others whose extracts and isolated pure natural products were considerably active [18]. Results of this study further support the anthelmintic potential of lichens as reported [8].

The cytotoxicity test revealed a negligible risk of all but one extract (hexane extract of *H. obscurata*), with CC_50_ > 30 *μ*g/mL [17]. However, only one (methanol extract of *P. tinctorum*) showed high selectivity for the adult male worm (Table 2). This may be due to quantitative (yield) and qualitative (chemical constituents) differences in the phytochemicals in *P. tinctorum* compared to the other lichens (Table 1). The relatively abundant phytochemicals (saponins, tannins, flavonoids, and steroids), detected (Table 1), may be responsible for the observed anthelmintic effect and should be isolated and characterized.

The two most active extracts against male and female worms did not cause death of treated mice with no adverse effects observed. As evidence of lack of adversity, there was no significant difference in activities of liver enzymes between the control and test groups (Figures 2 and 3). The AST : ALT ratio (0.54) for mice treated with hexane extract of *U. articulata* was less than 1 further indicating the absence of toxicity to the liver [19]. However, the high AST : ALT ratio of 2.59 for the methanol extract of *P. tinctorum* indicates damage to mitochondria in the mouse hepatocytes releasing high amounts of AST into the blood [19]. Since no mortality and signs of adverse acute effects were observed, it suggests the damage in the liver cells was likely reversible considering the high reserve capacity of the liver. The dose of 2000 mg/kg is much higher than the therapeutic dose of 10 mg/70 kg body for ivermectin used to treat onchocerciasis and is intended to reveal potential toxicity in humans at the highest safe dose. An acute toxicity study of *Stachytarpheta cayennensis* extract at higher doses (2500-5000 mg/kg) in rats reported high AST : ALT ratios > 3 with no mortality but significant histopathological changes compared to the control animals indicating liver injury [20]; this was considered as severe toxicity which could be lethal at much higher doses. Wang et al. [19] demonstrated increased AST : ALT ratio in carbon tetrachloride-induced hepatic injury with significant histopathological changes, increase in inflammatory and depletion of antioxidant biomarkers in mice compared to controls. All pathological changes were significantly reversed using pharmacological agents, suggesting the high AST : ALT ratio points to severe reversible injury which may be aggravated and lethal at higher doses. There is also considerable experimental evidence of hepatocyte proliferation following acetaminophen injury [21]; this may explain the no mortality outcome in the mice despite evidence of severe injury.

## 5. Conclusions

This study has revealed high activity and selectivity for the adult male *O. ochengi* worm, of the *P. tinctorum* found on Mount Cameroon; hence, it is a potential source of new macrofilaricides. The secondary metabolites in this lichen should be isolated for further chemical and anthelmintic characterization. The considerable activity of *U. articulata* against the female worm should also be further explored.

## Figures and Tables

**Table 1:** Yield and classes of secondary metabolites in lichen extracts.

Extract code	Yield (%)	Alkaloids	Cardiac glycosides	Flavonoid	Phenolics	Saponins	Steroids	Tanins
USa_met_	1.3	-	+	++	++	++	++	++
USa_hex_	1.4	++	+++	+	-	++	+++	-
Pam_met_	12.9	-	-	++	-	+++	+	+++
Pam_hex_	1.2	-	+	++	-	+	+++	++
Het_met_	1.1	-	+	+	-	+++	++	+
Het_hex_	6.2	-	-	+	-	++	+	-

Relative amounts of secondary metabolites: -, absent; +, trace; ++, moderate; +++, high. USa: *Usnea articulata*; Pam: *Parmotrema tinctorum*; Het: *Heterodermia obscurata*. Subscripts: hex = hexane extract; met = methanol extract.

**Table 2:** Cytotoxicity and selectivity indices of lichen extracts.

Extract code	CC_50_ (*μ*g/mL)	IC_50_ (*μ*g/mL)			IC_100_ (*μ*g/mL)			Selectivity index (CC_50_/IC_50_)		
	Cells	AM	AF	MF	AM	AF	MF	AM	AF	MF
USa_hex_	122.8	32.01	36.34	ND	62.5	125	ND	3.84	3.38	ND
USa_met_	126.8	32.61	41.26	101.8	62.5	125	125	3.89	3.07	1.25
Pam_hex_	85.59	52.43	64.42	ND	15.625	250	ND	1.63	1.33	ND
Pam_met_	171.9	8.059	77.64	ND	15.625	250	ND	21.33	2.21	ND
Het_hex_	23.97	14.48	72.74	ND	31.25	250	ND	1.66	0.33	ND
Het_met_	37.46	34.65	115.2	ND	125	250	ND	1.08	0.33	ND

Extract codes are defined in Table 1. MF = microfilariae; AM = adult male; AF = adult female; ND = not done.

## Data Availability

All data of this work are available from the corresponding author upon reasonable request.
